# A plea for symptom-based research in psychiatry

**DOI:** 10.3402/ejpt.v6.27660

**Published:** 2015-05-19

**Authors:** Ulrike Schmidt

**Affiliations:** RG Molecular Psychotraumatology, Clinical Department, Max Planck Institute of Psychiatry, Munich, Germany

**Keywords:** Posttraumatic stress disorder, subthreshold PTSD, subclinical PTSD, subsyndromal PTSD, symptom-based research, RDoC, PTSD subtypes, PTSD subtyping

## Abstract

**Background:**

The significant proportion of patients suffering from subthreshold diagnoses such as partial posttraumatic stress disorder (PTSD) shows that today's diagnostic entities do not fully meet the reality and needs of clinical practice. Moreover, as stated also in the recently announced concept of research domain criteria (RDoC), the use of today's traditional diagnostic systems in psychiatric research does not sufficiently promote an integrative understanding of mental disorders across multiple units of analysis from behavior to neurobiology. Besides RDoC, core symptom-based research concepts have been proposed to bridge the translational gap in psychiatry, but, unfortunately, have not yet become the rule.

**Objective/method:**

First, this article briefly reviews literature on subthreshold PTSD (as an example for subthreshold diagnoses) and, second, pleas for and proposes a modified symptom-based research concept in psychiatry.

**Results:**

Subthreshold PTSD has, like other subthreshold psychiatric diagnoses, not yet been clearly defined. Diagnostic entities such as subthreshold PTSD are subject to a certain arbitrariness as they are mainly the result of empiricism. This fact stresses the urgent need for neurobiologically-informed psychiatric diagnoses and motivated the here-presented proposal of a symptom-based research concept. As proposed here, and before by other researchers, symptom-based research in psychiatry should refrain from studying patient cohorts compiled according to diagnoses but, instead, should focus on assessing cohorts grouped according to chief complaints or predominant psychopathological symptoms.

**Conclusions:**

The linkage of the RDoC concept and symptom-based psychiatric research might probably speed up the definition of biologically or symptom-based psychiatric diagnoses, which might replace the auxiliary constructs of “traditional” diagnoses such as full and subthreshold PTSD, and promote the development of novel psychological and pharmacological treatments.

Traumatic experiences can result in the development of a variety of psychiatric diseases, for instance, in major depression (Chiu et al., 2011), panic disorder (Bryant, Creamer, O'Donnell, McFarlane, & Silove, 2014), and posttraumatic stress disorder (PTSD) (De Vries & Olff, 2009). PTSD has been defined as a psychiatric syndrome *inter alia* going along with aversive re-experiencing of the traumatic incident, nervous hyperarousal, emotional numbing, and avoidance anxiety (American Psychiatric Association, 2013; Cloitre, Garvert, Weiss, Carlson, & Bryant, 2014). However, a significant proportion of trauma-exposed individuals does not develop full but subthreshold PTSD (McLaughlin et al., 2015). Subthreshold PTSD might constitute a prodromal syndrome for PTSD and/or a potential secondary PTSD risk marker (for definition, see Schmidt et al., 2015) as it progresses to full PTSD in a number of affected individuals (Cukor, Wyka, Jayasinghe, & Difede, 2010). In general, the terms psychiatric subthreshold, subsyndromal, partial, or subclinical diagnosis are used synonymously and refer to a clinical syndrome that does not fully meet the DSM-5 (American Psychiatric Association, 2013) or ICD-10 (Graubner, 2014) diagnosis criteria of the respective mental disorder. In many trauma-exposed populations (Glück, Tran, & Lueger-Schuster, 2012; Muhtz et al., 2011; Rothenhäusler, 2006; Teegen & Meister, 2000; Van Zelst, De Beurs, Beekman, Deeg, & Van Dyck, 2003), but not in all (Frueh, Grubaugh, Yeager, & Magruder, 2009; Wagner, Müller, & Maercker, 2012), the subthreshold PTSD is more prevalent than full PTSD.

Traumatic experiences can result in the development of a variety of psychiatric diseases, for instance, in major depression (Chiu et al., 2011), panic disorder (Bryant, Creamer, O'Donnell, McFarlane, & Silove, 2014), and posttraumatic stress disorder (PTSD) (De Vries & Olff, 2009). PTSD has been defined as a psychiatric syndrome *inter alia* going along with aversive re-experiencing of the traumatic incident, nervous hyperarousal, emotional numbing, and avoidance anxiety (American Psychiatric Association, 2013; Cloitre, Garvert, Weiss, Carlson, & Bryant, 2014). However, a significant proportion of trauma-exposed individuals does not develop full but subthreshold PTSD (McLaughlin et al., 2015). Subthreshold PTSD might constitute a prodromal syndrome for PTSD and/or a potential secondary PTSD risk marker (for definition, see Schmidt et al., 2015) as it progresses to full PTSD in a number of affected individuals (Cukor, Wyka, Jayasinghe, & Difede, 2010). In general, the terms psychiatric subthreshold, subsyndromal, partial, or subclinical diagnosis are used synonymously and refer to a clinical syndrome that does not fully meet the DSM-5 (American Psychiatric Association, 2013) or ICD-10 (Graubner, 2014) diagnosis criteria of the respective mental disorder. In many trauma-exposed populations (Glück, Tran, & Lueger-Schuster, 2012; Muhtz et al., 2011; Rothenhäusler, 2006; Teegen & Meister, 2000; Van Zelst, De Beurs, Beekman, Deeg, & Van Dyck, 2003), but not in all (Frueh, Grubaugh, Yeager, & Magruder, 2009; Wagner, Müller, & Maercker, 2012), the subthreshold PTSD is more prevalent than full PTSD.

The “clinical presentation of subthreshold PTSD can vary widely and is, therefore, not clearly defined, nor is there an evidence-based treatment approach” tested in this population (Costanzo et al., 2014). However, several authors and study groups elaborated sophisticated definitions or diagnosis criteria for subthreshold PTSD: Kasckow, Yeager, and Magruder (2015) compared four definitions of subthreshold PTSD in primary care veterans and found them to be “nearly equivalent in their ability to discriminate individuals” (p. 43). Moreover, McLaughlin et al. (2015) examined over 20,000 respondents of The World Health Organization World Mental Health Surveys reporting prior trauma exposure in order to establish criteria for subthreshold PTSD—the latter study group concluded that “subthreshold DSM-5 PTSD is most usefully defined as meeting two or three of DSM-5 criteria B-E” (p. 375). Another research group aimed to identify subthreshold PTSD by psychophysiological measurements (Roy, Costanzo, & Leaman, 2012; Roy et al., 2013). The concept of partial/subthreshold PTSD was established in the 1990s (Bryant, 1996; McLaughlin et al., 2015; Schützwohl & Maercker, 1999). Today, reports on subthreshold PTSD are still sparse, but increased in number in the last years.

Besides PTSD, numerous other psychiatric syndromes can manifest in subclinical forms such as subthreshold depression (Baumeister, 2010; Cuijpers et al., 2014) and subthreshold generalized anxiety disorder (Haller, Cramer, Lauche, Gass, & Dobos, 2014). The awareness of the fact that subthreshold “psychiatric disorders constitute a major public health burden in terms of psychiatric morbidity among adolescents” (Roberts, Fisher, Blake Turner, & Tang, 2015, p. 397) and adults (Cohen, Magai, Yaffee, & Walcott-Brown, 2005; Haller et al., 2014) as well as the awareness of the significant amount of psychiatric patients resistant to available treatment options (Bondolfi et al., 1998; Dunlop, Kaye, Youngner, & Rothbaum, 2014; Zarate et al., 2004) stimulated psychological and biological subtyping of psychiatric disorders. In PTSD research, these efforts resulted *inter alia* in proposals of symptomatically (Flood et al., 2010; Spiegel et al., 2013; Weston, 2014) and biologically (Mehta et al., 2011; Zaba et al., 2015) distinct PTSD endophenotypes. The PTSD dissociative subtype has reached the level of a diagnosis in DSM-5 (American Psychiatric Association, 2013).

Altogether, the studies on subclinical psychiatric syndromes such as subthreshold PTSD have not yet resulted in generally accepted definitions. However, the increasing amount of studies on subthreshold mental disorders and the efforts to further refine psychiatric diagnoses reflect the insight of the medical scientific community that psychiatric diagnoses are auxiliary constructs that need to be optimized. As “a psychiatric diagnosis today is asked to serve many functions—clinical, research, medicolegal, delimiting insurance coverage, service planning, and defining eligibility for state benefits” (Herlihy & Turner, 2015; Szmukler, 2014, p. 517), generally accepted definitions of subthreshold psychiatric syndromes certainly will help the affected individuals, e.g., by promoting health insurance covering of their treatment. Unless there is no biologically routed diagnosis system available for mental disorders, the current ICD and DSM systems are without alternative—at least for clinical, medicolegal, and insurance issues. However, as the past has shown, it is very unlikely that the establishment of further refined diagnoses merely relating to phenotypical criteria promotes the enlightenment of the neurobiological basis of these diseases. Using the traditional diagnosis systems in neuro- and psychobiological research has hitherto not resulted in a significant diminution of the huge translational gap in psychiatry.

## From syndrome to symptom-based research in psychiatry?

For this reason, most neuropsychobiological researchers agree that future diagnostic systems for mental disorders should be based on their neurobiological characteristics and not on phenomenological descriptions. This agreement has resulted in proposals of alternative research concepts, *inter alia* in formulation of ROAMER (roadmap for mental health research in Europe) (Haro et al., 2014) and of the research domain criteria (RDoC). “The mandate for RDoC is to consider psychopathology in terms of maladaptive extremes along a continuum of normal functioning” (Ford et al., 2014). Rather than establishing pathophysiology associated with diagnoses of the DSM-5 and ICD-10 diagnosis catalogues, the RDoC approach encourages us to start analyzing physiological neural circuit functioning and healthy, adaptive behavioral *before* studying how alterations in these systems could lead to pathology (Ford et al., 2014). Thus, in the RDoC approach, the use of subthreshold and full psychiatric diagnoses is *quasi* obsolete. Instead, the center of RDoC is a matrix of functional dimensions, grouped into domains such as systems for social processes and cognition. Each of these domains can be studied using different classes of variables (termed “units of analysis”) of which eight were hitherto specified: genes, molecules, cells, neural circuits, physiology, behaviors, self-reports, and paradigms (webpage of the National Institute of Mental Health: www.nimh.nih.gov/research-priorities/rdoc/research-domain-criteria of RDoC: matrix.shtml). In summary, RDoC is a dimensional approach that relies on dimensions ranging from normal to pathological. Suggestions on how to study distinct psychopathological symptoms like hallucinations and anxiety using the RDoC approach have already been made (Ford et al., 2014; Simpson, 2012). To the best of our knowledge, the hitherto-only paper that tried to build a vision on how RDoC might transform the field of PTSD research is a manuscript highlighting the importance of conditioned fear associated paradigms for trauma and stress research (Briscione, Jovanovic, & Norrholm, 2014).

In this paper, a symptom-based research approach is suggested that, in contrast to RDoC, concentrates on dimensionally analyzing *pathological* conditions, but that, in agreement with RDoC, does not follow diagnoses defined in DSM-5 or ICD-10, nor any other diagnosis-oriented approach. Hence, the RDoC and the here-suggested symptom-based research approach might well complement each other.

## A symptom-based research concept in psychiatry

Years ago, other researchers suggested similar symptom-oriented research approaches (Fleeson, Furr, & Arnold, 2010; Sharpe & Walker, 2009) to bridge the evident translational gap in psychiatry, but unfortunately their concepts have not yet been implemented sufficiently in clinical psychiatric research: we still keep on analyzing the pathobiology of DSM and ICD diagnostic entities, usually by comparing cohorts of healthy controls with cohorts of patients fulfilling the criteria of a distinct DSM-5 or ICD-10 diagnosis.

The mandate for this here-proposed symptom-based concept is simple, namely to dimensionally analyze psychiatric symptoms that are either self-reported by affected individuals (e.g., depressed mood, non-suicidal self-injury, re-experiencing of traumatic events) or noted by an external observer (e.g., paraphrasing, cognitive symptoms) or recorded by a diagnostic instrument or procedure (e.g., hippocampal volume loss). Probably, the most challenging part of this approach is the compilation of the list of symptoms. In this list, which should constantly be expanded according to the persistently changing state of the literature, psychotrauma-related mental ailments could be represented by symptoms such as “aversive re-experiencing” and “increased startle reflex”—the latter has been repeatedly shown to discriminate in PTSD patients from healthy controls and from patients with other disorders (Schmidt, Kaltwasser, & Wotjak, 2013).

The detailed and dimensional assessment of psychopathological symptoms is a core feature of the here-proposed symptom-based research concept. In symptom-based psychiatric research, a structured interview for subjectively reported and externally observed psychopathological symptoms should replace structured interviews for the determination of DSM or ICD diagnoses. In a symptom-based interview, *all* psychopathological symptoms (and *not* only *subsets* of symptoms occurring in specific disorders) need to be quantified in intensity. For that purpose, currently available instruments allowing the dimensional assessment of psychopathological symptoms could be integrated. From the hitherto primarily used diagnosis-oriented approach in clinical psychiatry, the here-proposed symptom-based oriented approach mainly differs in the inclusion and the evaluation procedure: For symptom-based research, patients with *different diagnoses* should be included and, for evaluation, should be grouped according to the severity of psychopathological or psychophysiological symptoms and not, as done so far, according to syndromes/diagnoses. This procedure would allow detecting trajectories across diagnoses and might, hence, significantly enhance the chance to enlighten the biological underpinnings of psychopathology as psychopathological symptoms are more likely to share a common pathobiology than psychiatric diagnoses assembled by expert gremia according to epidemiological and phenomenological criteria. Hence, symptom-based research might, together with other approaches such as RDoC, be a chance to fundamentally increase our understanding of mental diseases thereby hopefully promoting the development of novel classes of psychotropic drugs. Symptom-based research could be integrated into the RDoC approach by defining psychopathological symptoms as “units of analysis” of the RDoC system. Fusing the RDoC and symptom-based psychiatric research approaches might result in biologically or symptom-based psychiatric diagnoses which, in turn, might replace the auxiliary constructs of the traditional full and subthreshold diagnoses.

A brief outline of how a fused symptom-based/RDoC could look like: characterizing a cohort of inpatients using a symptom-based research approach could result in identification of a cohort with extreme obsessive–compulsive symptoms, of another cohort with extreme avoidance anxiety, and again another cohort with extreme agitation. Of course, ICD and DSM diagnoses would differ among the individuals belonging to one and the same cohort as patients were not grouped according to the traditional diagnostic systems. In an intercohort analysis, patients suffering from an *extreme* expression of distinct symptoms could be compared to individuals showing a *low* expression of the respective symptoms. Alternatively, intracohort analyses testing the statistical correlation of symptom severity and another biological or psychological variable could be performed. In these procedures of inter- and intracohort analyses, units of analysis of the RDoC approach could be integrated—the RDoC units “genes,” “molecules,” and “neural circuits” seem to be particularly suitable for this approach. Evaluation of the resulting huge data sets comprising hundreds of psychopathological items and hundreds to thousands of molecular data could be performed in analogy to the well-established evaluation procedures of molecular microarrays.

Suggestions on how to apply RDoC in practice have already been made by several authors. In accordance with the here-proposed fusion of the symptom-based and RDoC approaches, several authors suggested RDoC approaches for *symptoms* and not for *syndromes or diagnoses*, *inter alia* for anxiety (Simpson, 2012), social anxiety (Fang, Hoge, Heinrichs, & Hofmann, 2014), hallucinations (Ford et al., 2014), and compulsivity/impulsivity (Berlin & Hollander, 2014). These suggestions might represent the dawn of a new era in psychiatry and should encourage us to leave the beaten paths.

## Supplementary Material

A plea for symptom-based research in psychiatry

A plea for symptom-based research in psychiatry

A plea for symptom-based research in psychiatry

A plea for symptom-based research in psychiatry

A plea for symptom-based research in psychiatry

A plea for symptom-based research in psychiatry
