# Supplementary material for: A plea for symptom-based research in psychiatry
Source: Eur J Psychotraumatol. 2015 May 19;6:10.3402/ejpt.v6.27660. doi: 10.3402/ejpt.v6.27660 (PMC4439426; doi:10.3402/ejpt.v6.27660)
Supplement: A plea for symptom-based research in psychiatry [file EJPT-6-27660-s001.pdf]

Plaidoyer pour une recherche fondée sur les symptômes en psychiatrie

Ulrike Schmidt

Contexte: Une proportion significative de patients souffrant de diagnostics infraliminaires comme l'ESPT partiel, montre que les actuelles entités de diagnostic ne satisfont pas entièrement la réalité et les besoins de la pratique clinique. En outre, comme indiqué également dans le concept récemment annoncé des critères de domaine de recherche (RDoC), l'utilisation des systèmes de diagnostic traditionnels dans la recherche psychiatrique ne favorise pas suffisamment une compréhension intégrée des troubles mentaux à travers de multiples unités d'analyse, du comportement à la neurobiologie. Outre RDoC, les concepts de recherche basés sur les symptômes ont été proposés pour combler le fossé translationnel en psychiatrie, mais, malheureusement, ne sont pas encore devenue la règle.
Objectif / Méthode: D'abord, cet article passe brièvement en revue la littérature sur l'ESPT infraliminaire (comme un exemple pour les diagnostics infraliminaires) et, deuxièmement, offre un plaidoyer et propose une modification du concept de recherche fondée sur les symptômes en psychiatrie.
Résultats: L'ESPT infraliminaire, comme les autres diagnostics psychiatriques infraliminaires, n'a pas encore été clairement défini. Les entités diagnostics comme l'ESPT infraliminaire sont soumis à un certain arbitraire car ils résultent principalement de l'empirisme. Ce fait souligne l'urgente nécessité pour les diagnostics psychiatriques dont la neurobiologie est assez bien connue et a motivé la proposition présentée ici d'un concept de recherche fondée sur les symptômes. Telle que proposée ici, et avant par d'autres chercheurs, la recherche fondée sur les symptômes en psychiatrie doit s'abstenir d'étudier des cohortes de patients recrutés selon leurs diagnostics, mais, au contraire, devrait se concentrer sur l'évaluation des cohortes regroupées selon les plaintes principales ou les symptômes psychopathologiques prédominants.
Conclusions: Le lien entre le concept de RDoC et de la recherche psychiatrique fondée sur les symptômes pourrait sans doute accélérer l'identification de diagnostics psychiatriques définis par leur biologie ou basé sur les symptômes, qui pourrait remplacer les constructions auxiliaires de diagnostics «traditionnels», comme les ESPT supra- et infraliminaires, et de promouvoir le développement de nouveaux traitements psychologiques et pharmacologiques.

Mots-clés: état de stress post-traumatique, ESPT, ESPT infraliminaire, ESPT infraclinique, ESPT subsyndromal, recherche fondée sur les symptômes, RDoC, sous-types d'ESPT, ESPT sous-typage

Citation: European Journal of Psychotraumatology 2015, **6**: 27660 - http://dx.doi.org/10.3402/ejpt.v6.27660
