# Supplementary material for: A plea for symptom-based research in psychiatry
Source: Eur J Psychotraumatol. 2015 May 19;6:10.3402/ejpt.v6.27660. doi: 10.3402/ejpt.v6.27660 (PMC4439426; doi:10.3402/ejpt.v6.27660)
Supplement: A plea for symptom-based research in psychiatry [file EJPT-6-27660-s002.pdf]

Ein Plädoyer für eine Symptom-orientierte psychiatrische Forschung

Ulrike Schmidt

Hintergrund: Der erhebliche Teil der Patienten mit subsyndromalen psychiatrischen Diagnosen wie beispielsweise der partiellen Posttraumatischen Belastungsstörung (PTBS) zeigt, dass die heutzutage verwendeten psychiatrischen Diagnosen nicht der Realität und den Bedürfnissen der klinischen Praxis entsprechen. Wie auch im Zusammenhang mit dem kürzlich veröffentlichten Forschungskonzept RDOC (*engl.* Research Domain Criteria) festgestellt wurde, wird das integrative Verständnis der Grundlagen psychischer Störungen durch die Verwendung traditioneller Diagnosesysteme nicht hinreichend gefördert. Mit dem Ziel, psychiatrische Forschung grundlegend zu verbessern, entwickelten einige Autoren Symptom-orientierte Forschungskonzepte – leider haben sich diese neuen Konzepte aber bislang noch nicht durchgesetzt. Klinische psychiatrische Forschung besteht weiterhin überwiegend aus Studien, die Kohorten von Pateinten die die Kriterien bestimmter traditioneller Diagnosen erfüllen, mit Kohorten von psychisch gesunden Individuen vergleichen. Hieraus resultierten zwar eine Fülle von Erkenntnissen über psychische Störungen im Allgemeinen, jedoch noch keine neuartigen Therapiemethoden.
Ziel / Methode: Auf einen kurzen Überblick Literatur über die partielle PTBS (als Beispiel für subsyndromale Diagnosen) erfolgt ein Plädoyer für die Einführung eines Symptom-orientierten Forschungskonzepts in der Psychiatrie, das an Beispielen erläutert wird.
Ergebnisse: Die subsyndromale PTBS ist, wie alle anderen subsydromalen psychiatrischen Diagnosen auch, noch nicht eindeutig definiert. Die Definition von Diagnose-Einheiten wie der partiellen PTBS beruht in erster Linie auf empirischen Erkenntnissen und unterliegt damit einer gewissen Willkür. Dieser Umstand unterstreicht die dringende Notwendigkeit, neurobiologisch fundierte psychiatrische Diagnosen zu etablieren und motiviert das hier präsentierte Symptom-orientierte Forschungskonzept. Wie hier, und zuvor bereits von anderen Forschern, vorgeschlagen wird, sollten die zu untersuchenden Patientenkohorten in der Symptom-basierten Forschung nicht nach bestimmten traditionellen psychiatrischen Diagnosen, sondern nach Hauptbeschwerden oder dominierenden psychopathologischen Symptomen gruppiert werden. Dieses Vorgehen macht die Diagnose- bzw. Erkrankungs-übergreifende Untersuchung großer Patientengruppen mit einem detaillierten dimensionalen Symptom-Diagnosekatalog erforderlich und die Störungs-spezifische Analyse psychopathologischer Symptome obsolet.
Schlussfolgerung: Da es viel wahrscheinlicher ist, die biologische Ursache distinkter psychopathologischer *Symptome* als die empirisch zusammengestellter psychiatrischer *Syndrome* zu erfolgreich zu entschlüsseln, würde die Fusion des RDOC Konzepts und des Symptom-orientierten Forschungskonzepts den Erkenntnisprozess der psychiatrischen Forschung vermutlich deutlich beschleunigen. Es ist anzunehmen, dass dadurch die Definition neuartiger neurobiologisch-fundierter psychiatrischer Diagnosen und damit auch die Entwicklung neuer Behandlungsverfahren nachhaltig gefördert werden würde.

Schlüsselwörter: Posttraumatische Belastungsstörung, PTBS, unterschwellige PTBS, subklinische PTBS, subsyndromale PTBS, symptom-basierte Forschung, RDoC, PTBS-Subtypen, PTBS-Subtypisierung

Citation: European Journal of Psychotraumatology 2015, **6**: 27660 - http://dx.doi.org/10.3402/ejpt.v6.27660
