# Supplementary material for: A plea for symptom-based research in psychiatry
Source: Eur J Psychotraumatol. 2015 May 19;6:10.3402/ejpt.v6.27660. doi: 10.3402/ejpt.v6.27660 (PMC4439426; doi:10.3402/ejpt.v6.27660)
Supplement: A plea for symptom-based research in psychiatry [file EJPT-6-27660-s003.pdf]

Érvek a tünetalapú pszichiátriai kutatások mellett

Ulrike Schmidt

Háttér: A küszöbalatti PTSD-vel diagnosztizálható betegek magas száma azt mutatja, hogy a jelenleg hatályban lévő diagnosztikus eszközök nem teljes mértékben találkoznak a valósággal és a klinikai szükségletekkel. Továbbá, ahogy a „research domain criteria (RDoC)"-ban leírták, a tradicionális diagnosztikus rendszerek a pszichiátriai kutatásban nem segítik elő a mentális betegségek, integratív szemléletét átívelve a viselkedéstől a neurobiológiáig. Az RDoc-on kívül, javasolták már a tünetalapú kutatást, a kutatás és a gyakorlat közötti hézag kiküszöbölésére, de mégsem ez a gyakorlat.
Célkitűzés/módszer: Először a küszöbalatti PTSD-ről szóló irodalom kerül áttekintésre (mint egy példa a küszöbalatti diagnózisokra), majd érvek kerülnek bemutatásra a tünetalapú pszichiátriai kutatás mellett.
Eredmények: A küszöbalatti PTSD, akárcsak más hasonló diagnózis, még nincs pontosan definiálva. Mivel az ilyen diagnózisok főleg tapasztalati alapon kerülnek kialakításra, így ki vannak téve önkényes döntéseknek. Éppen ezért is fontos a neurobiológiai alapokon nyugvó pszichiátriai diagnózisalkotás és a tüneteken alapuló kutatás. Ahogy itt és máshol is leírták, a tünetalapú kutatás lényege az, hogy nem diagnosztikus kategóriák alapján, hanem vezető panaszok és túlsúlyban lévő pszichopatológiai tünetek alapján vizsgálják a betegeket.
Következtetések: Az RDoc elképzelés és a tünetalapú kutatás összekötése felgyorsíthatja a biológiai vagy tünetalapú diagnózis alkalmazását, ami felválthatná a tradicionális diagnosztikus kategóriákat és elősegítheti új pszichológiai és gyógyszeres kezelések fejlődését.

Kulcsszavak: poszttraumás stressz zavar; PTSD; küszöbalatti PTSD; szubklinikus PTSD; szubszindromális PTSD; tünetalapú kutatás; RDoC; PTSD altípusok; PTSD altipizálás

Citation: European Journal of Psychotraumatology 2015, **6**: 27660 - http://dx.doi.org/10.3402/ejpt.v6.27660
