# Supplementary material for: A plea for symptom-based research in psychiatry
Source: Eur J Psychotraumatol. 2015 May 19;6:10.3402/ejpt.v6.27660. doi: 10.3402/ejpt.v6.27660 (PMC4439426; doi:10.3402/ejpt.v6.27660)
Supplement: A plea for symptom-based research in psychiatry [file EJPT-6-27660-s004.pdf]

Una llamada a la investigación psiquiátrica basada en el síntoma

Ulrike Schmidt

Antecedentes: La significativa proporción de pacientes que sufren de diagnósticos subumbrales como el TEPT parcial muestra que las entidades de diagnóstico de hoy en día no encajan plenamente con la realidad y las necesidades de la práctica clínica. Por otra parte, como se indica también en el concepto recientemente anunciado de criterios del campo de investigación (RDoC, por las siglas en inglés de *research domain criteria*), el uso de los actuales sistemas de diagnóstico tradicionales en la investigación psiquiátrica no fomenta lo suficiente la comprensión integradora de los trastornos mentales a través de las numerosas unidades de análisis desde el comportamiento a la neurobiología. Además de los RDoC, se han propuesto conceptos centrales de investigación basados en los síntomas para cerrar la brecha translacional en psiquiatría, pero, por desgracia, *aún no se han normativizado*.
Objetivo / Método: En primer lugar, este artículo revisa brevemente la literatura sobre el TEPT subumbral (como ejemplo de diagnóstico subumbral) y, segundo, solicita y propone un concepto modificado de investigación psiquiátrica basado en los síntomas.
Resultados: El trastorno de estrés postraumático subumbral, al igual que otros diagnósticos psiquiátricos subumbrales, todavía no ha sido definido claramente. Las entidades diagnósticas como el TEPT subumbral están sujetas a una cierta arbitrariedad, ya que son principalmente el resultado del empirismo. Este hecho pone de relieve la necesidad urgente de diagnósticos psiquiátricos informados neurobiológicamente y ha motivado la propuesta del concepto de investigación basado en los síntomas que aquí presentamos. Tal y como proponemos aquí, y como han hecho antes otros investigadores, la investigación psiquiátrica basada en los síntomas debería abstenerse de estudiar cohortes de pacientes compiladas en función de los diagnósticos y, en cambio, debería centrarse en la evaluación de cohortes agrupadas de acuerdo con las quejas principales o los síntomas psicopatológicos predominantes.
Conclusiones: El vínculo entre el concepto de la RDoC y la investigación psiquiátrica basada en los síntomas probablemente pueda acelerar la definición de los diagnósticos psiquiátricos basados en los síntomas o en la biología que puedan sustituir a los constructos auxiliares de diagnósticos "tradicionales" como el de TEPT completo y subumbral y fomentar el desarrollo de nuevos *tratamientos farmacológicos* y psicológicos.

Palabras clave: Trastorno de estrés postraumático, TEPT, TEPT subumbral, TEPT subclínico, TEPT subsindrómico, investigación basada en los síntomas, RDoC, subtipos de TEPT

Citation: European Journal of Psychotraumatology 2015, **6**: 27660 - http://dx.doi.org/10.3402/ejpt.v6.27660
