# Supplementary material for: A plea for symptom-based research in psychiatry
Source: Eur J Psychotraumatol. 2015 May 19;6:10.3402/ejpt.v6.27660. doi: 10.3402/ejpt.v6.27660 (PMC4439426; doi:10.3402/ejpt.v6.27660)
Supplement: A plea for symptom-based research in psychiatry [file EJPT-6-27660-s005.pdf]

**Psikiyatride semptom temelli araştırma için bir talep**

Ulrike Schmidt

Arkaplan: Kısmi TSSB gibi eşikaltı tanılardan muzdarip hastaların önemli bir kısmı bugünün tanısal kavramlarının gerçekliği ve klinik pratik ihtiyaçlarını tam olarak karşılamadığını göstermektedir. Bunun yanında, yakın zamanda duyurulan araştırma alanı kriterleri konseptinde (RDoC) olduğu gibi, psikiyatrik araştırmada bugünün geleneksel tanısal sistemlerinin davranıştan nörobiyolojiye çoklu analiz birimleri boyunca mental bozuklukların bütünleyici şekilde anlaşılmasını yeterince desteklememektedir. RDoC ötesinde, semptom temelli temel araştırma konseptleri psikiyatrideki çeviriyle ilgili boşluk için köprü kurmak amacıyla önerilmiş, fakat, ne yazık ki, henüz kural haline gelmemiştir.
Amaç/Metod: İlk olarak, bu makale kısaca eşikaltı TSSB ( eşikaltı tanılar için bir örnek olarak) üzerine olan literatürü gözden geçirmektedir, ve, ikincisi, psikiyatrideki değiştirilmiş semptom temelli araştırma konsepti talebinde ve önerisinde bulunmaktadır.
Sonuçlar: Eşikaltı TSSB, diğer eşikaltı psikiyatrik tanılar gibi, henüz açıkça tanımlanmamıştır. Eşikaltı TSSB gibi tanısal kavramlar ampirizmin ana şekildeki sonuçları gibiymişçesine belli nedensizliklere maruz kalırlar. Bu durum, semptom temelli araştırma konseptinin gerekçeli mevcut önerisini ve nörobiyolojik olarak bilgilendirici psikiyatrik tanılara acil ihtiyacı vurgulamaktadır. Burada, ve daha önce diğer araştırmacılar tarafından, önerildiği gibi, psikiyatrideki semptom temelli araştırmalar tanılara göre oluşturulan hasta grupları ile çalışmaktan kaçınmalı, onun yerine, en baskın psikopatolojik semptomları ya da ana şikayetlere göre ayrıştırılmış grupları değerlendirme üzerine odaklanmalıdırlar.
Tartışma: RDoC konsepti ve belirti temelli psikiyatrik araştırmalar bağlantısı tam ve eşikaltı TSSB gibi "geleneksel" tanıların yardımcı yapıları ile yer değiştirecek semptom temelli psikiyatrik tanıların ya da biyoloji olarak tanımları hızlandırabilir ve yeni psikolojik ve farmakolojik tedavilerin gelişimini destekleyebilir.

Anahtar Kelimeler: travma sonrası stres bozukluğu, TSSB, eşikaltı TSSB, subklinik TSSB, subsendromal TSSB, semptom temelli araştırma, RDoC, TSSB alttip, TSSB alttipleme

**Citation:** European Journal of Psychotraumatology 2015, **6**: 27660 - http://dx.doi.org/10.3402/ejpt.v6.27660
