# Supplementary material for: A plea for symptom-based research in psychiatry
Source: Eur J Psychotraumatol. 2015 May 19;6:10.3402/ejpt.v6.27660. doi: 10.3402/ejpt.v6.27660 (PMC4439426; doi:10.3402/ejpt.v6.27660)
Supplement: A plea for symptom-based research in psychiatry [file EJPT-6-27660-s006.pdf]

Molba za istraživanja u psihijatriji bazirana na simptomima

Ulrike Schmidt

Uvod: Značajan procenat pacijenata koji pate od dijagnoze "ispod dijagnostičkog praga" kao što je parcijalni PTSP ukazuje da današnji dijagnostički entiteti ne odgovaraju u potpunosti stvarnosti i potrebama kliničke prakse. Čak šta više, kao što je navedeno u skoro objavljenom konceptu kriterijuma istraživačkih domena (concept of research domain criteria-RDoC), današnja upotreba tradicionalnih dijagnostičkih sistema u psihijatrijiskim istraživanjima nedovoljno unapređuje integrativno razumevanje mentalnih poremećaja kroz multiple jedinice analize, od bihejvora do neurobiologije. Pored RDoC, koncept istraživanja koja se baziraju na ključnim simptomima je predložen da premosti psihijatrijski jaz, ali, na žalost, *još uvek nije postao pravilo.*
Cilj/Metod: Prvo, ovo je kratak pregled literature o PTSP-u "ispod praga dijagnostičkih kriterijuma" (kao primeru takve dijagnoze) i, drugo, ukazuje na i predlaže modifikovani koncept istraživanja u psihijatriji koji se zasniva na simptomima.
Rezultati: PTSP "ispod dijagnostičkog praga" (subthreshold), kao i drugi slični poremećaji, još uvek nije jasno definisan. Dijagnostički entiteti kao što su subthreshold PTSP su predmet rasprava s obzirom da se zasnivaju na empirizmu. Ova činjenica naglašava hitnu potrebu za psihijatrijskom dijagnozom na osnovu neurobioloških podataka i motiviše za ovde-prezentovani predlog koncepta istraživanja koji se zasniva na simptomima. Kao što je ovde predloženo, i pre drugih istraživača, ovakva vrsta istraživanja u psihijatriji bi promenila proučavanje kohorte pacijenata grupisanih prema dijagnozi u proučavanje kohorte grupisane prema glavnim tegobama ili predominantnim psihopatološkim simptomima.
Zaključak: Veza RDoC koncepta i psihijatrijskih istraživanja baziranih na simptomima verovatno bi mogla ubrzati definisanje bioloških ili na simptomima zasnovanih psihijatrijskih dijagnoza koje bi mogle zameniti pomoćne konstruke "tradicionalne" dijagnoze kao potpuni i subthreshold PTSP i unaprediti razvoj novih psiholoških i farmakoloških tretmana.

Ključne reči: posttraumatski stresni poremećaj, PTSP, subthreshold PTSP, subklinički PTSP, subsindromalni PTSP, istraživanja zasnovana na simptomima, RdoC, podtip PTSP-a

Citation: European Journal of Psychotraumatology 2015, **6**: 27660 - http://dx.doi.org/10.3402/ejpt.v6.27660
